# Crystal structure of bis­{3-(3,4-di­meth­oxy­phen­yl)-5-[6-(pyrazol-1-yl)pyridin-2-yl]-1,2,4-triazol-3-ato}iron(II)–methanol–chloro­form (1/2/2)

**DOI:** 10.1107/S2056989023008423

**Published:** 2023-09-29

**Authors:** Kateryna Znovjyak, Igor O. Fritsky, Tatiana Y. Sliva, Vladimir M. Amirkhanov, Sergey O. Malinkin, Sergiu Shova, Maksym Seredyuk

**Affiliations:** a Department of Chemistry, Taras Shevchenko National University of Kyiv, Volodymyrska Street 64, Kyiv, 01601, Ukraine; b Department of Inorganic Polymers, "Petru Poni" Institute of Macromolecular, Chemistry, Romanian Academy of Science, Aleea Grigore Ghica Voda 41-A, Iasi, 700487, Romania; University of Neuchâtel, Switzerland

**Keywords:** crystal structure, iron(II) complexes, neutral complexes

## Abstract

The unit cell of the title compound, [Fe(C_18_H_15_N_6_O_2_)_2_]·2CH_3_OH·2CHCl_3_, consists of a charge-neutral complex mol­ecule, two methanol and two chloro­form mol­ecules. In the complex, the two tridentate 2-(5-(3,4-di­meth­oxy­phen­yl)-1,2,4-triazol-3-yl)-6-(pyrazol-1-yl)pyridine ligands coordinate to the central Fe^II^ ion through the N atoms of the pyrazole, pyridine and triazole groups, forming a pseudo-octa­hedral coordination sphere. Neighbouring tapered mol­ecules are linked through weak C—H(pz)⋯π(ph) inter­actions into one-dimensional chains, which are joined into two-dimensional layers through weak C—H⋯N/C/O inter­actions. Furthermore, the layers stack in a three-dimensional network linked by weak inter­layer C—H⋯π inter­actions of the meth­oxy and phenyl groups. The inter­molecular contacts were qu­anti­fied using Hirshfeld surface analysis and two-dimensional fingerprint plots, revealing the relative contributions of the contacts to the crystal packing to be H⋯H 32.0%, H⋯C/C⋯H 26.3%, H⋯N/N⋯H 13.8%, and H⋯O/O⋯H 7.5%. The average Fe—N bond distance is 2.185 Å, indicating the high-spin state of the Fe^II^ ion. Energy framework analysis at the HF/3–21 G theory level was performed to qu­antify the inter­action energies in the crystal structure.

## Abstract 2

The title compound, a charge-neutral bis­{5-(3,4-di­meth­oxy­phen­yl)-1,2,4-triazol-3-ato)-6-(pyrazol-1-yl)pyridine}iron(II) di­methanol di­chloro­form solvate, is a high-spin complex with a distorted pseudo­octa­hedral coordination environment of the metal ion. Due to the tapered shape and polar nature, the mol­ecules stack in one-dimensional columns that are bound by weak hydrogen bonds into layers, which, in turn, are arranged in a three-dimensional network without inter­layer inter­actions below van der Waals radii.

## 1. Chemical context

A broad class of coordination compounds exhibiting spin-state switching between low- (total spin *S* = 0) and high-spin states (total spin *S* = 2) is represented by Fe^II^ complexes based on tridentate bis­azole­pyridine ligands (Halcrow, 2014 ▸; Suryadevara *et al.*, 2022 ▸; Halcrow *et al.*, 2019 ▸). In the case of asymmetric ligand design, where one of the azole groups carries a hydrogen on a nitro­gen heteroatom and acts as a Brønsted acid, deprotonation can produce neutral complexes that can be either high spin (Schäfer *et al.*, 2013 ▸) or low spin (Shiga *et al.*, 2019 ▸) or exhibit temperature-induced transition between the spin states of the central atom (Seredyuk *et al.*, 2014 ▸; Grunwald *et al.*, 2023 ▸) depending on the ligand field strength. The periphery of the mol­ecule, *i.e*. ligand substituents, also plays an important role in the behaviour, determining the way that mol­ecules are packed in the crystal and their inter­actions with each other, and therefore further influencing the spin state adopted by the central atom. For example, the dynamic rearrangement of the meth­oxy group between bent and extended configurations can lead to a highly hysteretic spin transition *via* a supra­molecular blocking mechanism (Sered­yuk *et al.*, 2022 ▸). 
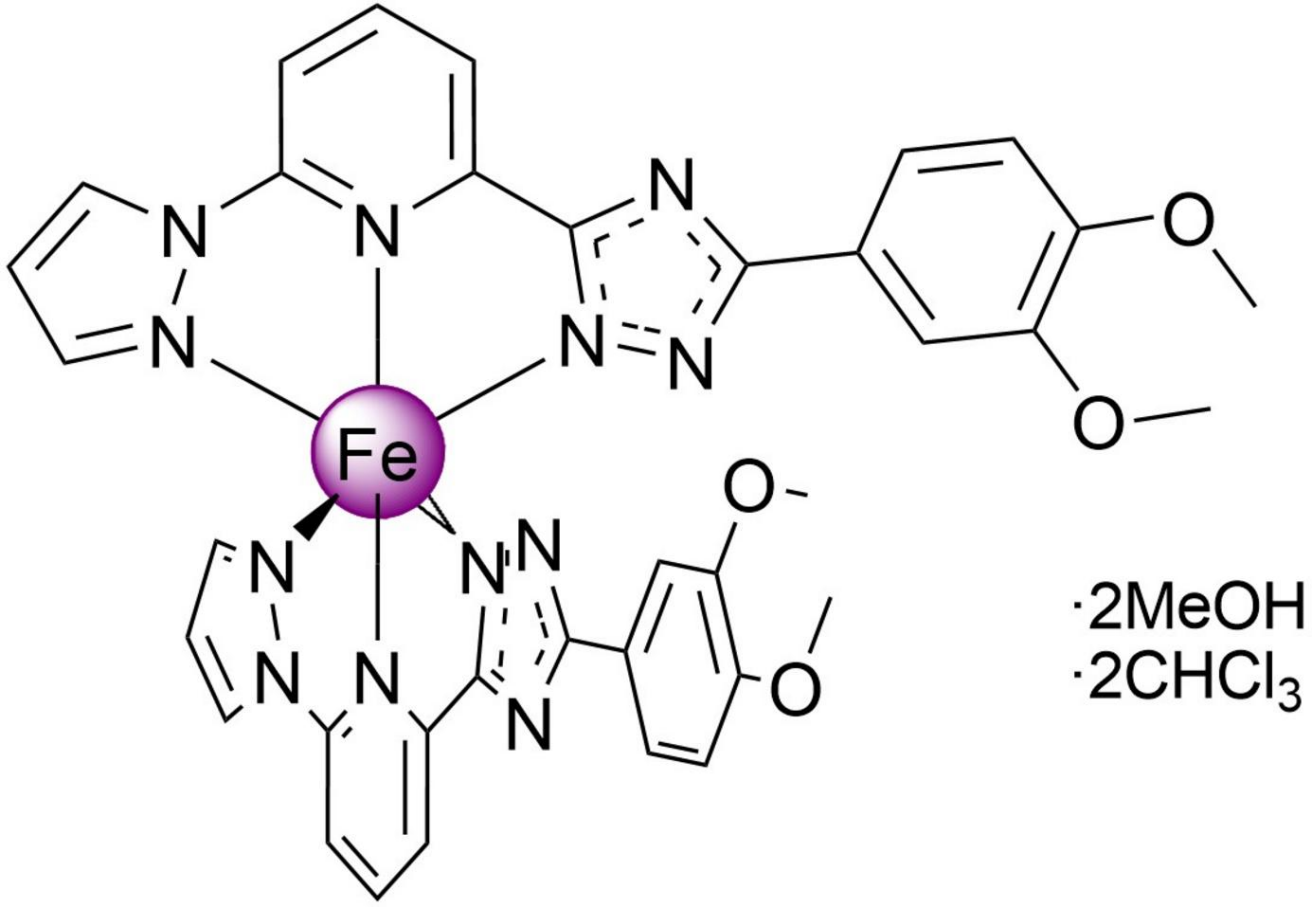


Having inter­est in spin-transition 3*d*-metal complexes formed by polydentate ligands (Bartual-Murgui *et al.*, 2017 ▸; Bonhommeau *et al.*, 2012 ▸; Valverde-Muñoz *et al.*, 2020 ▸), we report here a new [Fe^II^ *L*_2_] complex based on the asymmetric deprotonable ligand with two substituents on the phenyl group, *L* = 2-(5-(3,4-di­meth­oxy­phen­yl)-1,2,4-triazol-3-yl)-6- (pyrazol-1-yl)pyridine.

## 2. Structural commentary

**Figure 1:**
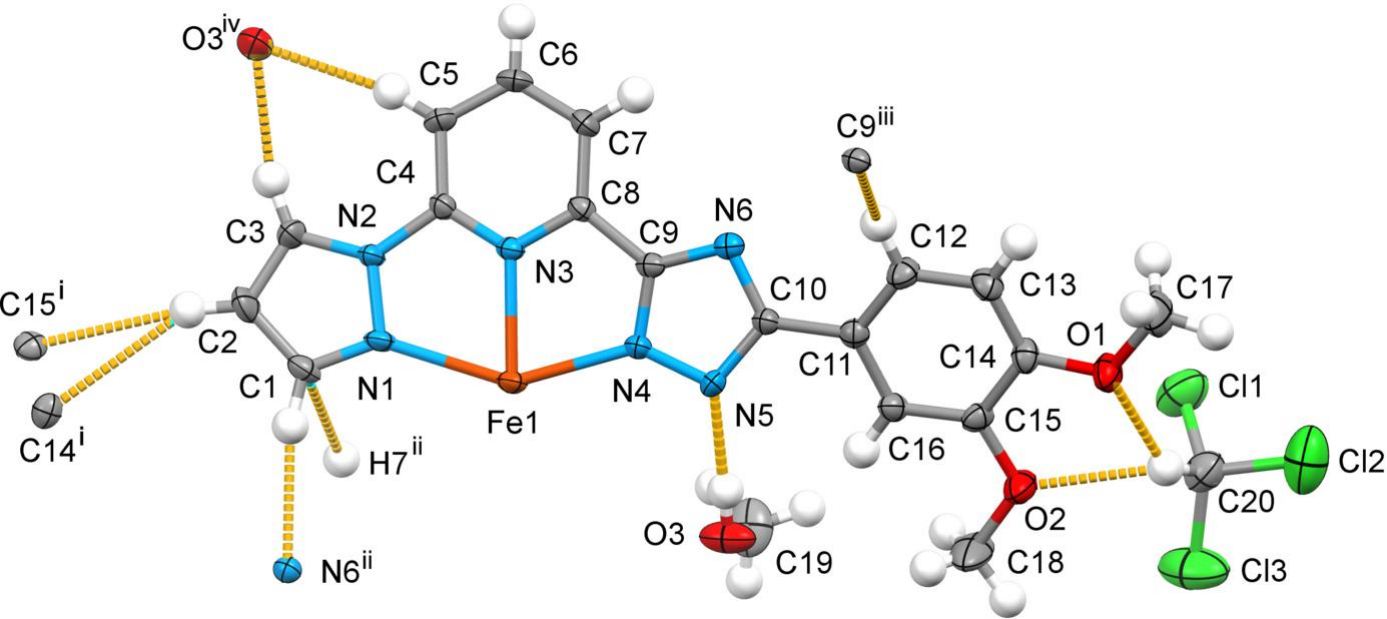
The mol­ecular structure of a half of the title compound with displacement ellipsoids drawn at the 50% probability level. The strong O—H⋯N and weak C—H⋯O/N/C hydrogen bonds are shown with the nearest neighbours. Symmetry codes: (i) 1 − *x*, 1 + *y*, 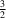 − *z*; (ii) − 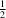 + *x*, 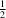 + *y*, 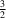 − *z*; (iii) 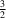 − *x*, − 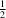 + *y*, *z*; (iv) 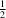 + *x*, 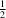 + *y*, 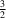 − *z*.

The complex has a tapered structure with divergent phenyl groups. The ligand mol­ecules are almost planar, including the meth­oxy substituents, which are also in the plane of the phenyl group. The independent methanol mol­ecule forms O—H⋯N hydrogen bonds with the triazole (trz) rings of the ligand mol­ecule (Fig. 1 ▸, Table 1 ▸). The chloro­form mol­ecules form double weak C—H⋯O bonds with the meth­oxy groups of the ligand. The central Fe^II^ ion of the complex has a distorted octa­hedral N_6_ coordination environment formed by the nitro­gen donor atoms of two tridentate ligands (Fig. 1 ▸). The average bond length, <Fe—N> = 2.185 Å, is typical for high-spin complexes with an N_6_ coordination environment (Gütlich & Goodwin, 2004 ▸). The average trigonal distortion parameters *Σ* = Σ_1_^12^(|90 − *φ*_i_|), where *φ*_i_ is the angle N—Fe—N′ (Drew *et al.*, 1995 ▸), and *Θ* = Σ_1_^24^(|60 − *θ*_i_|), where *θ*_i_ is the angle generated by superposition of two opposite faces of an octa­hedron (Chang *et al.*, 1990 ▸) are 148.6 and 474.2°, respectively. The values reveal a deviation of the coordination environment from an ideal octa­hedron (where *Σ* = *Θ* = 0), which is, however, in the expected range for bis­azole­pyridine and similar ligands (see below). The calculated continuous shape measure (CShM) value relative to the ideal *O_h_* symmetry is 5.391 (Kershaw Cook *et al.*, 2015 ▸). The volume of the [FeN_6_] coordination polyhedron is 12.796 Å^3^.

## 3. Supra­molecular features

**Figure 2:**
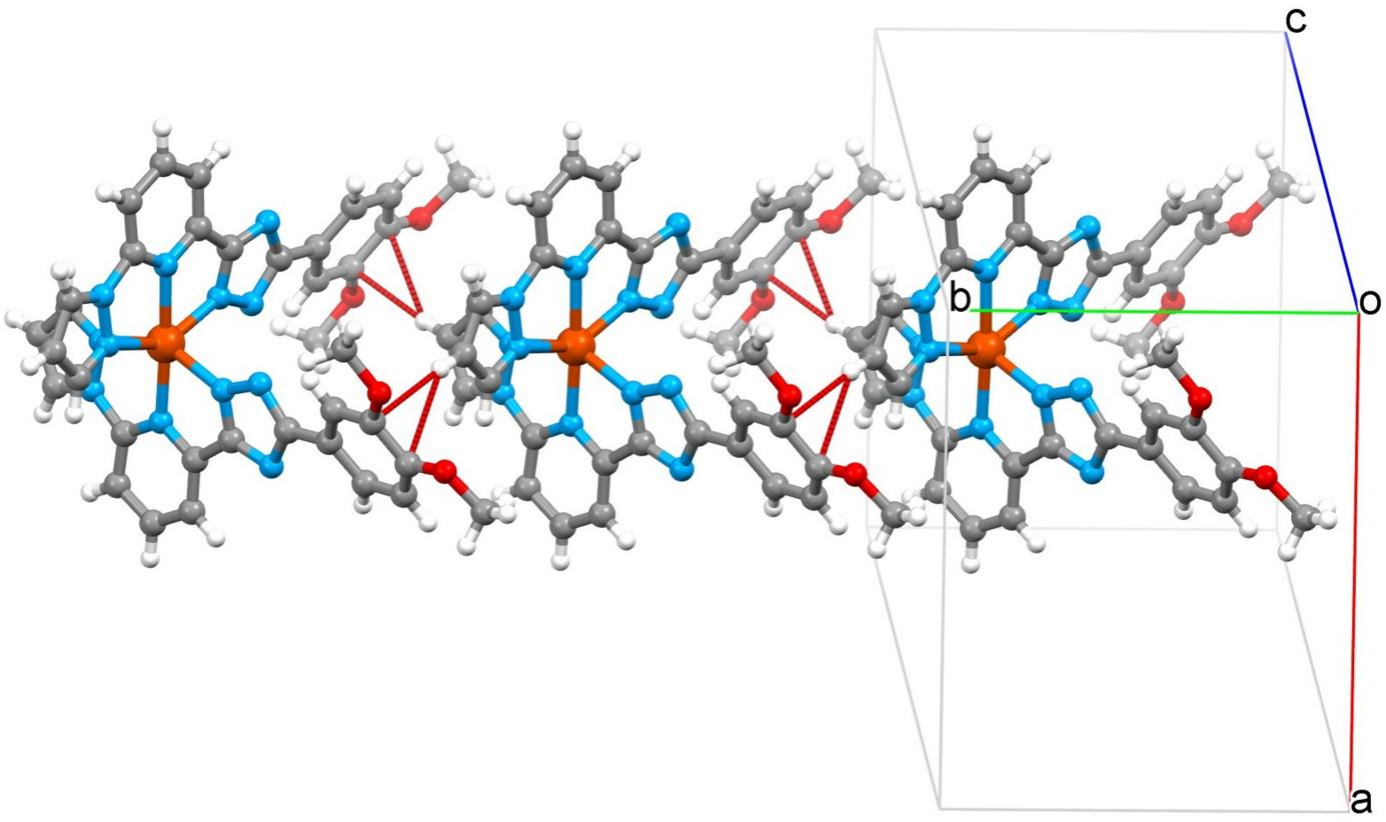
One-dimensional supra­molecular chain formed by stacking mol­ecules of the title compound. Red dashed lines correspond to contacts between the pyrazole and phenyl groups of neighbouring mol­ecules below the sum of van der Waals radii.

Due to the tapered structure, neighbouring complex mol­ecules fit into each other and inter­act through a weak C—H(pz)⋯π(ph) inter­molecular contact between the pyrazole (pz) and phenyl (ph) groups respectively [the C2⋯*Cg*(ph) distance is 3.574 (5) Å]. The one-dimensional supra­molecular chains formed extend along the *b-*axis direction with the stacking periodicity equal to 10.281 (3) Å (= cell parameter *b*) (Fig. 2 ▸)*.* Through weak inter­molecular C—H(pz, py)⋯ N/C(pz, trz) inter­actions in the range 3.115–3.705 (5) Å (Table 1 ▸), neighbouring chains are joined into corrugated two-dimensional layers in the *ab* plane (Fig. S1*a*,*b* in the supporting information). The layers stack without any inter­layer inter­actions below the van der Waals radii (Fig. S1*b* in the supporting information). The voids between the layers are occupied by solvent mol­ecules, which also participate in the bonding within separate layers. The methanol mol­ecule forms a strong O—H⋯N hydrogen bond with the deprotonated triazole group, and a chloro­form mol­ecule located between two meth­oxy groups of the phenyl substituent forms a five-membered cyclic motif with two C—H⋯O bonds (see Fig. 1 ▸). A complete list of the considered inter­molecular inter­actions is given in Table 1 ▸.

## 4. Hirshfeld surface and 2D fingerprint plots

**Figure 3:**
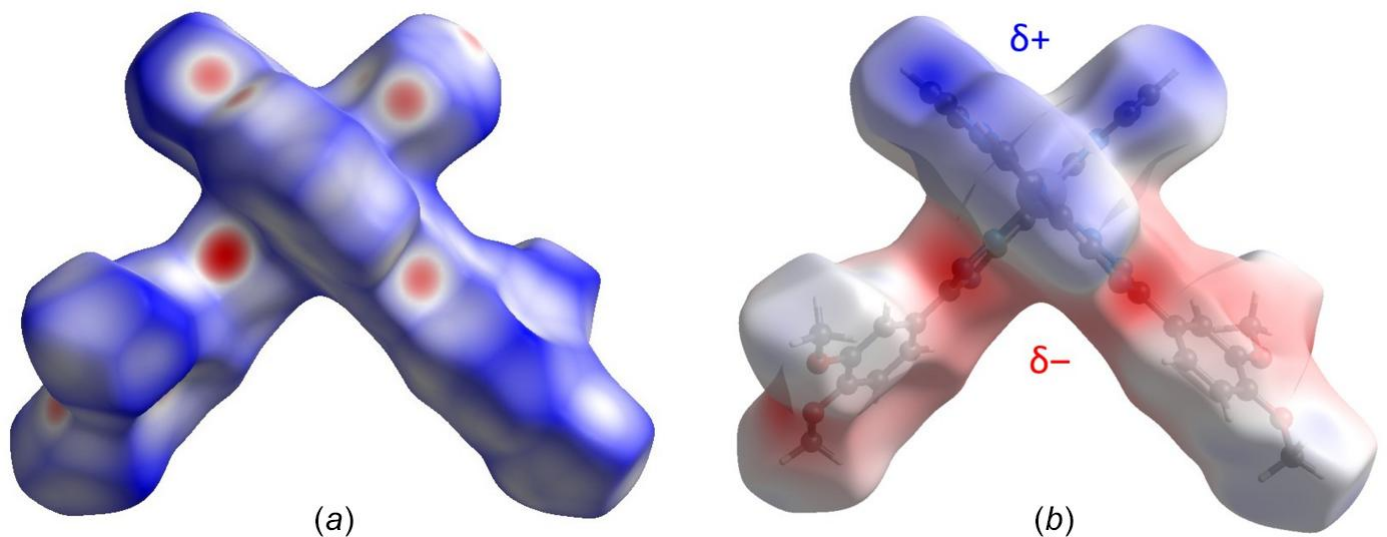
(*a*) A projection of *d*_norm_ mapped on the Hirshfeld surfaces, showing the inter­molecular inter­actions within the mol­ecule. Red/blue and white areas represent regions where contacts are shorter/larger than the sum and close to the sum of the van der Waals radii, respectively. (*b*) Electrostatic potential for the title compound derived from a HF/3–21 G wavefunction mapped on the Hirshfeld surface in the range −0.1658 (red) to 0.1235 a.u. (blue).

**Figure 4:**
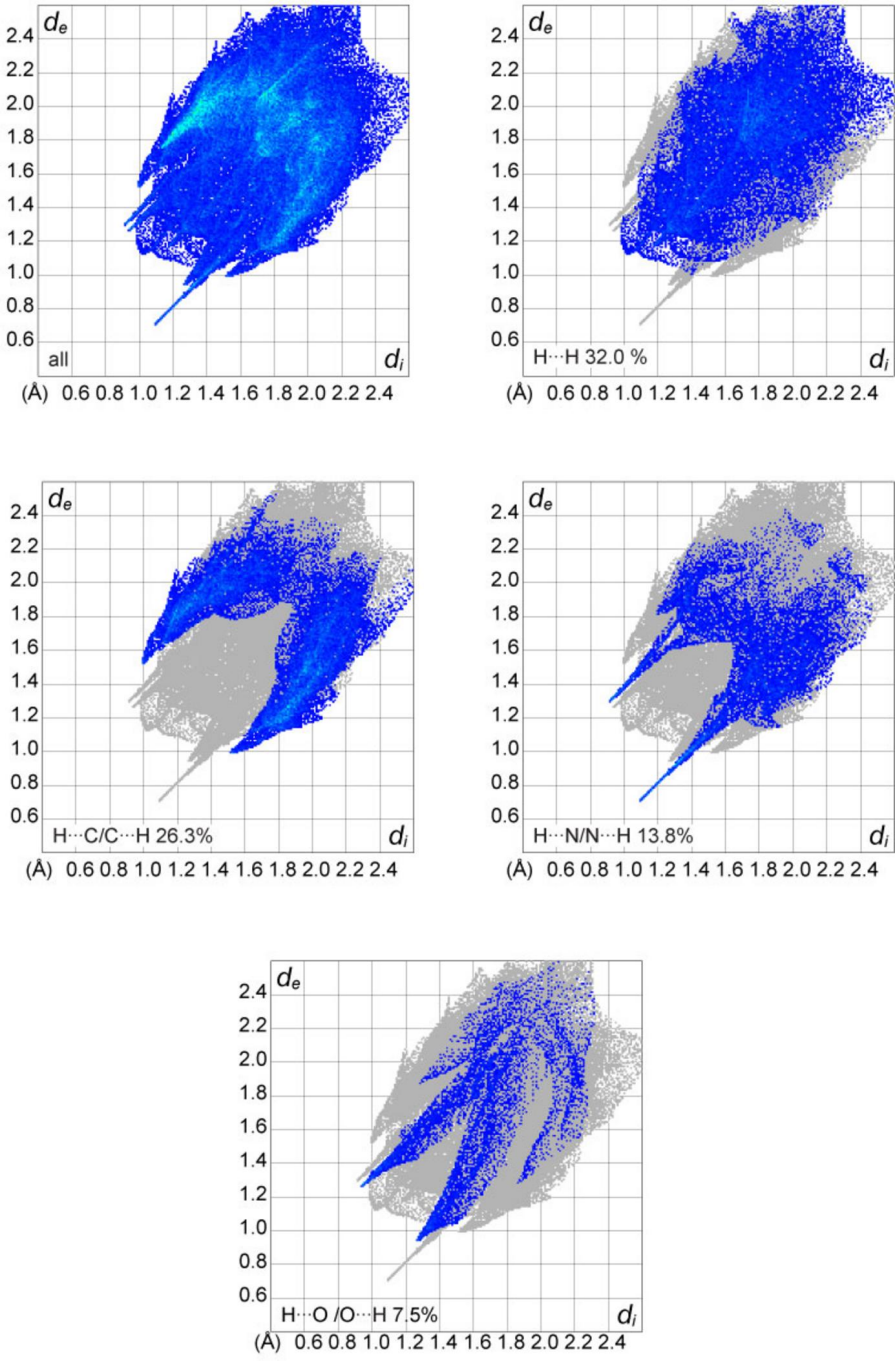
The overall two-dimensional fingerprint plot and those decomposed into specified inter­actions.

Hirshfeld surface analysis was performed and the associated two-dimensional fingerprint plots were generated using *CrystalExplorer* (Spackman *et al.*, 2021 ▸), with a standard resolution of the three-dimensional *d*_norm_ surfaces plotted over a fixed colour scale of −0.6492 (red) to 1.3918 (blue) a.u. (Fig. 3 ▸ *a*). The pale-red spots symbolize short contacts and negative *d*_norm_ values on the surface corresponding to the inter­actions described above. The overall two-dimensional fingerprint plot is illustrated in Fig. 4 ▸. The two-dimensional fingerprint plots, with their relative contributions to the Hirshfeld surface, are shown for the H⋯H, H⋯C/C⋯H, H⋯N/N⋯H and H⋯O/O⋯H contacts together with the . At 32.0%, the largest contribution to the overall crystal packing is from H⋯H inter­actions, which are located in the middle region of the fingerprint plot. H⋯C/C⋯H contacts contribute 26.3% to the Hirshfeld surface and result in a pair of characteristic wings. The H⋯N/N⋯H contacts, represented by a pair of sharp spikes in the fingerprint plot, make a 13.8% contribution to the Hirshfeld surface. Finally, H⋯O/O⋯H contacts, which account for a 7.5% contribution, are mostly distributed in the middle part of the plot. The electrostatic potential energy calculated using the HF/3-21G basis set localizes the negative charge on the trz-ph moieties of the complex mol­ecule, while the pz-py moieties are relatively positively charged (Fig. 3 ▸ *b*). The polar nature of the mol­ecule justifies the stacking in columns.

## 5. Energy framework analysis

The energy framework (Spackman *et al.*, 2021 ▸), calculated using the wave function at the HF/3-21G theory level, including the electrostatic potential forces (*E*_ele_), the dispersion forces (*E*_dis_) and the total energy diagrams (*E*_tot_), is shown in Fig. S2 in the supporting information. The cylindrical radii, adjusted to the same scale factor of 100, are proportional to the relative strength of the corresponding energies. The major contribution is due to the dispersion forces (*E*_dis_), reflecting dominating inter­actions in the crystal of the neutral asymmetric mol­ecules. The topology of the energy framework resembles the topology of the inter­actions within and between the layers described above. The calculated value E_tot_ for the intra­chain inter­action is −57.2 kJ mol^−1^ and for inter­chain inter­actions are down to −114.6 kJ mol^−1^. The inter­layer inter­action energies are close to zero. The colour-coded inter­action mappings within a radius of 5.0 Å of a central reference mol­ecule for the title compound together with full details of the various contributions to the total energy (*E*_ele_, *E*_pol_, *E*_dis_, *E*_rep_) are shown in the table in Figure S2.

## 6. Database survey

A search of the Cambridge Structural Database (CSD, Version 5.42, last update February 2021; Groom *et al.*, 2016 ▸) reveals several similar neutral Fe^II^ complexes with a deprotonable azole group, for example, derivatives of a pyrazole­pyridine­tetra­zole, IGERIX and LUTGEO (Gentili *et al.*, 2015 ▸; Senthil Kumar *et al.*, 2015 ▸) and pyrazole-pyridine-benzimidazole XODCEB (Shiga *et al.*, 2019 ▸). In addition, there are related complexes based on phenathroline­tetra­zole, such as QIDJET (Zhang *et al.*, 2007 ▸), phenanthroline-benzimidazole, DOMQUT (Seredyuk *et al.*, 2014 ▸), di­pyridyl­pyrrol, NIRLOT (Grunwald *et al.*, 2023 ▸). The Fe—N distances of these complexes in the low-spin state are 1.933–1.959 Å, while in the high-spin state they are in the range 2.179–2.185 Å. The values of the trigonal distortion and CShM(*O_h_*) change correspondingly, and in the low-spin state they are systematically lower than in the high-spin state. Table 2 ▸ collates the structural parameters of the complexes and of the title compound.

## 7. Synthesis and crystallization

The synthesis of the title compound is identical to that reported recently for a similar complex (Seredyuk *et al.*, 2022 ▸). It was produced by using a layering technique in a standard test tube. The layering sequence was as follows: the bottom layer contained a solution of [Fe(*L*_2_)](BF_4_)_2_ prepared by dissolving *L* = 2-[5-(3,4-di­meth­oxy­phen­yl)-1,2,4-triazol-3-yl]-6-(pyrazol-1-yl)pyridine (88 mg, 0.252 mmol) and Fe(BF_4_)_2_·6H_2_O (43 mg, 0.126 mmol) in boiling acetone, to which chloro­form (5 ml) was then added. The middle layer was a methanol–chloro­form mixture (1:10, 10 ml), which was covered by a layer of methanol (10 ml), to which 100 ml of NEt_3_ were added dropwise. The tube was sealed, and black–orange single crystals appeared after 3–4 weeks (yield *ca* 60%). Elemental analysis calculated for C_40_H_40_Cl_6_FeN_12_O_6_: C, 45.61; H, 3.83; N, 15.96. Found: C, 45.52; H, 3.77; N, 15.77.

## 8. Refinement

Crystal data, data collection and structure refinement details are summarized in Table 3 ▸. H atoms were refined as riding [C—H = 0.95–0.98 Å with *U*_iso_(H) = 1.2–1.5*U*_eq_(C)]. The O-bound H atom was refined with *U*_iso_(H) = 1.5*U*_eq_(O).

## Supplementary Material

Crystal structure: contains datablock(s) I. DOI: 10.1107/S2056989023008423/tx2075sup1.cif

Structure factors: contains datablock(s) I. DOI: 10.1107/S2056989023008423/tx2075Isup2.hkl

Supporting information file. DOI: 10.1107/S2056989023008423/tx2075Isup3.cdx

Packing drawings and energy framework analysis data and drawings. DOI: 10.1107/S2056989023008423/tx2075sup5.doc

CCDC reference: 2297496

Additional supporting information:  crystallographic information; 3D view; checkCIF report

## Figures and Tables

**Table 1:** Hydrogen-bond geometry (Å, °)

*D*—H⋯*A*	*D*—H	H⋯*A*	*D*⋯*A*	*D*—H⋯*A*
C2—H2⋯C14^i^	0.95	2.85	3.676 (5)	146
C2—H2⋯C15^i^	0.95	2.64	3.585 (5)	171
C7—H7⋯C1^ii^	0.95	2.81	3.705 (5)	158
C1—H1⋯N6^iii^	0.95	2.34	3.267 (5)	166
C12—H12⋯C9^iv^	0.95	2.85	3.541 (5)	130
C20—H20⋯O1	1.00	2.28	3.115 (6)	141
C20—H20⋯O2	1.00	2.39	3.179 (6)	135
O3—H3*A*⋯N5	0.84	1.94	2.775 (4)	177
C3—H3⋯O3^v^	0.95	2.33	3.238 (5)	161
C5—H5⋯O3^v^	0.95	2.47	3.401 (6)	167

Symmetry codes: (i) 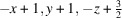 ; (ii) 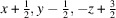 ; (iii) 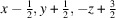 ; (iv) 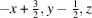 ; (v) 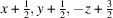 .

**Table 2:** Computed distortion indices (Å, °) for the title compound and for similar complexes reported in the literature

CSD Code	Spin state	<Fe—N>	Σ	Θ	CShM(*O_h_*)
Title compound	High-spin	2.185	148.6	474.2	5.39
IGERIX	High spin	2.179	149.7	553.2	6.06
IGERIX01	Low spin	1.986	105.6	350.6	2.85
LUTGEO	Low spin	1.933	85.0	309.6	2.10
XODCEB	Low spin	1.950	87.4	276.6	1.93
DOMQIH	Low spin	1.962	83.8	280.7	2.02
QIDJET01	Low spin	1.970	90.3	341.3	2.47
QIDJET	High spin	2.184	145.5	553.3	5.88
DOMQUT	Low spin	1.991	88.5	320.0	2.48
DOMQUT02	High spin	2.183	139.6	486.9	5.31
NIRLOT	Low spin	1.939	77.3	255.6	1.68

**Table 3:** Experimental details

Crystal data	
Chemical formula	[Fe(C_18_H_15_N_6_O_2_)_2_]·2CH_4_O·2CHCl_3_
*M*_r_	1053.39
Crystal system, space group	Orthorhombic, *P* *b* *c* *n*
Temperature (K)	180
*a*, *b*, *c* (Å)	12.7195 (9), 10.281 (3), 36.735 (3)
*V* (Å^3^)	4804.0 (13)
*Z*	4
Radiation type	Mo *K*α
μ (mm^−1^)	0.71
Crystal size (mm)	0.25 × 0.2 × 0.03

Data collection	
Diffractometer	Xcalibur, Eos
Absorption correction	Multi-scan (*CrysAlis PRO*; Rigaku OD, 2022 ▸)
*T*_min_, *T*_max_	0.995, 1.000
No. of measured, independent and observed [*I* > 2σ(*I*)] reflections	18857, 5510, 2962
*R*_int_	0.092
(sin θ/λ)_max_ (Å^−1^)	0.688

Refinement	
*R*[*F* ^2^ > 2σ(*F* ^2^)], *wR*(*F* ^2^), *S*	0.079, 0.145, 1.03
No. of reflections	5510
No. of parameters	298
H-atom treatment	H-atom parameters constrained
Δρ_max_, Δρ_min_ (e Å^−3^)	0.39, −0.44

Computer programs: *CrysAlis PRO* (Rigaku OD, 2022 ▸), *SHELXT* (Sheldrick, 2015*a* ▸), *SHELXL2018/3* (Sheldrick, 2015*b* ▸) and *OLEX2* (Dolomanov *et al.*, 2009 ▸).

## supplementary crystallographic information

### Crystal data

**Table:**

[Fe(C_18_H_15_N_6_O_2_)_2_]·2CH_4_O·2CHCl_3_	*D*_x_ = 1.456 Mg m^−^^3^
*M**_r_* = 1053.39	Mo *K*α radiation, λ = 0.71073 Å
Orthorhombic, *P**b**c**n*	Cell parameters from 2694 reflections
*a* = 12.7195 (9) Å	θ = 2.0–22.1°
*b* = 10.281 (3) Å	µ = 0.71 mm^−^^1^
*c* = 36.735 (3) Å	*T* = 180 K
*V* = 4804.0 (13) Å^3^	Prism, clear light yellow
*Z* = 4	0.25 × 0.2 × 0.03 mm
*F*(000) = 2160	

### Data collection

**Table:**

Xcalibur, Eos diffractometer	5510 independent reflections
Radiation source: fine-focus sealed X-ray tube, Enhance (Mo) X-ray Source	2962 reflections with *I* > 2σ(*I*)
Graphite monochromator	*R*_int_ = 0.092
Detector resolution: 16.1593 pixels mm^-1^	θ_max_ = 29.3°, θ_min_ = 2.0°
ω scans	*h* = −17→14
Absorption correction: multi-scan (CrysAlisPro; Rigaku OD, 2022)	*k* = −11→13
*T*_min_ = 0.995, *T*_max_ = 1.000	*l* = −30→45
18857 measured reflections	

### Refinement

**Table:**

Refinement on *F*^2^	0 restraints
Least-squares matrix: full	Hydrogen site location: inferred from neighbouring sites
*R*[*F*^2^ > 2σ(*F*^2^)] = 0.079	H-atom parameters constrained
*wR*(*F*^2^) = 0.145	*w* = 1/[σ^2^(*F*_o_^2^) + (0.0352*P*)^2^ + 1.3579*P*] where *P* = (*F*_o_^2^ + 2*F*_c_^2^)/3
*S* = 1.03	(Δ/σ)_max_ < 0.001
5510 reflections	Δρ_max_ = 0.39 e Å^−^^3^
298 parameters	Δρ_min_ = −0.44 e Å^−^^3^

### Special details

**Table:**

Geometry. All esds (except the esd in the dihedral angle between two l.s. planes) are estimated using the full covariance matrix. The cell esds are taken into account individually in the estimation of esds in distances, angles and torsion angles; correlations between esds in cell parameters are only used when they are defined by crystal symmetry. An approximate (isotropic) treatment of cell esds is used for estimating esds involving l.s. planes.

### Fractional atomic coordinates and isotropic or equivalent isotropic displacement parameters (Å^2^)

**Table:**

	*x*	*y*	*z*	*U*_iso_*/*U*_eq_	
Fe1	0.500000	0.76448 (9)	0.750000	0.0222 (2)	
Cl1	0.66889 (10)	0.30702 (16)	0.48491 (4)	0.0718 (5)	
Cl3	0.45098 (10)	0.2575 (2)	0.46990 (4)	0.0951 (7)	
Cl2	0.60243 (13)	0.05279 (18)	0.46193 (4)	0.0862 (6)	
N3	0.6647 (2)	0.7695 (3)	0.76381 (8)	0.0212 (8)	
O1	0.6486 (2)	0.1131 (3)	0.56399 (8)	0.0363 (8)	
N4	0.5728 (2)	0.6285 (3)	0.71371 (8)	0.0221 (8)	
N2	0.6252 (2)	0.9304 (3)	0.80493 (9)	0.0236 (8)	
N5	0.5447 (2)	0.5444 (3)	0.68666 (8)	0.0223 (8)	
O2	0.4939 (2)	0.2752 (3)	0.56650 (8)	0.0427 (9)	
N1	0.5214 (2)	0.9131 (3)	0.79464 (9)	0.0246 (8)	
O3	0.3669 (2)	0.6194 (4)	0.64823 (9)	0.0525 (10)	
H3A	0.421091	0.594475	0.659283	0.079*	
N6	0.7165 (2)	0.5103 (3)	0.69940 (8)	0.0221 (8)	
C9	0.6755 (3)	0.6051 (4)	0.72010 (10)	0.0216 (10)	
C8	0.7306 (3)	0.6878 (4)	0.74638 (10)	0.0208 (9)	
C14	0.6502 (3)	0.1976 (4)	0.59249 (11)	0.0272 (10)	
C4	0.7027 (3)	0.8539 (4)	0.78750 (10)	0.0222 (10)	
C16	0.5594 (3)	0.3751 (4)	0.62214 (11)	0.0261 (10)	
H16	0.501808	0.433833	0.623005	0.031*	
C3	0.6331 (3)	1.0150 (4)	0.83276 (12)	0.0348 (12)	
H3	0.696474	1.042065	0.844178	0.042*	
C12	0.7199 (3)	0.2910 (4)	0.64749 (11)	0.0296 (11)	
H12	0.773273	0.291996	0.665593	0.036*	
C7	0.8379 (3)	0.6927 (4)	0.75259 (11)	0.0295 (11)	
H7	0.884266	0.634916	0.740344	0.035*	
C10	0.6324 (3)	0.4768 (4)	0.67851 (10)	0.0222 (10)	
C5	0.8097 (3)	0.8673 (4)	0.79519 (11)	0.0300 (11)	
H5	0.835528	0.930144	0.811906	0.036*	
C1	0.4682 (3)	0.9884 (4)	0.81718 (11)	0.0281 (11)	
H1	0.393795	0.996704	0.816911	0.034*	
C13	0.7257 (3)	0.2015 (4)	0.61954 (11)	0.0304 (11)	
H13	0.782566	0.141665	0.618878	0.036*	
C11	0.6377 (3)	0.3795 (4)	0.64968 (10)	0.0246 (10)	
C2	0.5343 (3)	1.0548 (5)	0.84167 (13)	0.0399 (13)	
H2	0.514382	1.114171	0.860259	0.048*	
C15	0.5664 (3)	0.2858 (4)	0.59409 (11)	0.0266 (10)	
C6	0.8757 (3)	0.7825 (5)	0.77677 (12)	0.0361 (12)	
H6	0.949305	0.786965	0.781044	0.043*	
C20	0.5675 (3)	0.1923 (5)	0.48712 (13)	0.0490 (14)	
H20	0.556297	0.166973	0.513117	0.059*	
C17	0.7283 (3)	0.0157 (5)	0.56289 (13)	0.0488 (14)	
H17A	0.720138	−0.036527	0.540761	0.073*	
H17B	0.721896	−0.040564	0.584307	0.073*	
H17C	0.797583	0.057357	0.562860	0.073*	
C19	0.3952 (4)	0.7063 (6)	0.62079 (14)	0.0644 (17)	
H19A	0.433569	0.659748	0.601683	0.097*	
H19B	0.440099	0.774643	0.630986	0.097*	
H19C	0.331711	0.745638	0.610407	0.097*	
C18	0.4062 (4)	0.3632 (6)	0.56791 (15)	0.085 (2)	
H18A	0.357693	0.343789	0.547915	0.127*	
H18B	0.431588	0.452834	0.565572	0.127*	
H18C	0.369588	0.353055	0.591206	0.127*	

### Atomic displacement parameters (Å^2^)

**Table:**

	*U*^11^	*U*^22^	*U*^33^	*U*^12^	*U*^13^	*U*^23^
Fe1	0.0131 (4)	0.0258 (5)	0.0278 (4)	0.000	−0.0003 (3)	0.000
Cl1	0.0667 (9)	0.0745 (13)	0.0741 (11)	−0.0242 (8)	−0.0098 (7)	−0.0051 (10)
Cl3	0.0540 (9)	0.1473 (19)	0.0841 (12)	0.0051 (10)	−0.0120 (7)	0.0380 (14)
Cl2	0.1297 (13)	0.0655 (12)	0.0634 (11)	−0.0138 (11)	0.0108 (9)	−0.0116 (11)
N3	0.0149 (15)	0.023 (2)	0.0261 (18)	0.0011 (15)	−0.0012 (13)	−0.0012 (18)
O1	0.0454 (17)	0.029 (2)	0.0346 (18)	0.0121 (16)	−0.0062 (14)	−0.0080 (17)
N4	0.0150 (15)	0.026 (2)	0.0250 (19)	−0.0001 (15)	0.0003 (13)	−0.0049 (18)
N2	0.0133 (15)	0.023 (2)	0.034 (2)	0.0000 (14)	0.0004 (14)	−0.0089 (19)
N5	0.0204 (16)	0.024 (2)	0.0228 (18)	−0.0025 (15)	−0.0025 (13)	−0.0074 (18)
O2	0.0406 (17)	0.044 (2)	0.0439 (18)	0.0137 (16)	−0.0179 (14)	−0.0162 (18)
N1	0.0110 (15)	0.027 (2)	0.036 (2)	−0.0004 (14)	−0.0007 (13)	−0.0012 (19)
O3	0.0282 (15)	0.074 (3)	0.056 (2)	−0.0040 (18)	−0.0061 (15)	0.027 (2)
N6	0.0194 (16)	0.024 (2)	0.0232 (19)	−0.0008 (15)	−0.0015 (13)	0.0019 (17)
C9	0.0167 (19)	0.026 (3)	0.022 (2)	−0.0009 (18)	−0.0007 (15)	0.002 (2)
C8	0.0186 (19)	0.019 (2)	0.024 (2)	0.0022 (17)	0.0017 (16)	0.000 (2)
C14	0.035 (2)	0.022 (3)	0.025 (2)	0.004 (2)	0.0003 (18)	−0.001 (2)
C4	0.0177 (19)	0.023 (3)	0.026 (2)	−0.0007 (18)	0.0036 (16)	−0.003 (2)
C16	0.022 (2)	0.020 (3)	0.036 (3)	0.0009 (18)	−0.0020 (17)	−0.004 (2)
C3	0.024 (2)	0.034 (3)	0.046 (3)	−0.004 (2)	−0.0011 (19)	−0.019 (3)
C12	0.031 (2)	0.028 (3)	0.030 (2)	0.003 (2)	−0.0083 (18)	−0.001 (2)
C7	0.019 (2)	0.034 (3)	0.035 (2)	0.0060 (18)	0.0008 (17)	−0.010 (3)
C10	0.0180 (19)	0.023 (3)	0.025 (2)	−0.0001 (18)	0.0001 (16)	−0.002 (2)
C5	0.0190 (19)	0.034 (3)	0.037 (3)	−0.006 (2)	−0.0029 (18)	−0.008 (2)
C1	0.0181 (19)	0.026 (3)	0.040 (3)	0.0038 (18)	0.0061 (18)	0.000 (2)
C13	0.032 (2)	0.026 (3)	0.033 (3)	0.008 (2)	−0.0017 (18)	0.000 (2)
C11	0.024 (2)	0.024 (3)	0.025 (2)	−0.0024 (19)	0.0018 (17)	0.000 (2)
C2	0.032 (2)	0.035 (3)	0.053 (3)	0.006 (2)	0.009 (2)	−0.020 (3)
C15	0.026 (2)	0.023 (3)	0.031 (2)	−0.0042 (19)	−0.0047 (17)	−0.002 (2)
C6	0.0129 (19)	0.051 (4)	0.045 (3)	0.000 (2)	−0.0015 (18)	−0.011 (3)
C20	0.052 (3)	0.054 (4)	0.041 (3)	−0.008 (3)	−0.005 (2)	−0.002 (3)
C17	0.060 (3)	0.036 (3)	0.051 (3)	0.014 (3)	−0.003 (2)	−0.017 (3)
C19	0.096 (4)	0.051 (4)	0.047 (3)	0.007 (3)	0.001 (3)	0.013 (4)
C18	0.061 (3)	0.100 (6)	0.094 (5)	0.049 (4)	−0.051 (3)	−0.051 (5)

### Geometric parameters (Å, º)

**Table:**

Fe1—N3	2.156 (3)	C4—C5	1.398 (5)
Fe1—N3^i^	2.156 (3)	C16—H16	0.9500
Fe1—N4^i^	2.142 (3)	C16—C11	1.420 (5)
Fe1—N4	2.142 (3)	C16—C15	1.383 (5)
Fe1—N1	2.258 (3)	C3—H3	0.9500
Fe1—N1^i^	2.258 (3)	C3—C2	1.363 (5)
Cl1—C20	1.749 (5)	C12—H12	0.9500
Cl3—C20	1.745 (5)	C12—C13	1.380 (5)
Cl2—C20	1.764 (5)	C12—C11	1.389 (5)
N3—C8	1.348 (4)	C7—H7	0.9500
N3—C4	1.320 (5)	C7—C6	1.369 (5)
O1—C14	1.361 (5)	C10—C11	1.458 (5)
O1—C17	1.425 (5)	C5—H5	0.9500
N4—N5	1.365 (4)	C5—C6	1.386 (5)
N4—C9	1.349 (4)	C1—H1	0.9500
N2—N1	1.385 (4)	C1—C2	1.408 (6)
N2—C4	1.414 (4)	C13—H13	0.9500
N2—C3	1.346 (5)	C2—H2	0.9500
N5—C10	1.347 (4)	C6—H6	0.9500
O2—C15	1.374 (4)	C20—H20	1.0000
O2—C18	1.437 (5)	C17—H17A	0.9800
N1—C1	1.320 (5)	C17—H17B	0.9800
O3—H3A	0.8400	C17—H17C	0.9800
O3—C19	1.394 (5)	C19—H19A	0.9800
N6—C9	1.342 (5)	C19—H19B	0.9800
N6—C10	1.362 (4)	C19—H19C	0.9800
C9—C8	1.465 (5)	C18—H18A	0.9800
C8—C7	1.385 (5)	C18—H18B	0.9800
C14—C13	1.382 (5)	C18—H18C	0.9800
C14—C15	1.401 (5)		
			
N3^i^—Fe1—N3	177.28 (19)	C11—C12—H12	119.3
N3—Fe1—N1	72.28 (11)	C8—C7—H7	120.7
N3^i^—Fe1—N1	105.79 (11)	C6—C7—C8	118.5 (4)
N3^i^—Fe1—N1^i^	72.29 (11)	C6—C7—H7	120.7
N3—Fe1—N1^i^	105.79 (11)	N5—C10—N6	113.2 (4)
N4^i^—Fe1—N3	106.79 (11)	N5—C10—C11	123.6 (3)
N4—Fe1—N3^i^	106.79 (11)	N6—C10—C11	123.1 (3)
N4^i^—Fe1—N3^i^	75.05 (12)	C4—C5—H5	122.3
N4—Fe1—N3	75.05 (12)	C6—C5—C4	115.4 (4)
N4—Fe1—N4^i^	98.53 (18)	C6—C5—H5	122.3
N4^i^—Fe1—N1^i^	147.30 (10)	N1—C1—H1	123.9
N4^i^—Fe1—N1	92.40 (12)	N1—C1—C2	112.3 (3)
N4—Fe1—N1	147.30 (10)	C2—C1—H1	123.9
N4—Fe1—N1^i^	92.40 (12)	C14—C13—H13	119.4
N1—Fe1—N1^i^	94.81 (17)	C12—C13—C14	121.2 (4)
C8—N3—Fe1	118.5 (3)	C12—C13—H13	119.4
C4—N3—Fe1	121.7 (2)	C16—C11—C10	120.5 (4)
C4—N3—C8	119.7 (3)	C12—C11—C16	117.8 (4)
C14—O1—C17	117.3 (3)	C12—C11—C10	121.7 (3)
N5—N4—Fe1	138.9 (2)	C3—C2—C1	104.6 (4)
C9—N4—Fe1	115.3 (3)	C3—C2—H2	127.7
C9—N4—N5	105.5 (3)	C1—C2—H2	127.7
N1—N2—C4	118.0 (3)	O2—C15—C14	115.3 (4)
C3—N2—N1	111.2 (3)	O2—C15—C16	124.0 (4)
C3—N2—C4	130.7 (3)	C16—C15—C14	120.6 (4)
C10—N5—N4	105.8 (3)	C7—C6—C5	121.9 (3)
C15—O2—C18	116.3 (3)	C7—C6—H6	119.1
N2—N1—Fe1	113.6 (2)	C5—C6—H6	119.1
C1—N1—Fe1	142.2 (2)	Cl1—C20—Cl2	109.8 (2)
C1—N1—N2	104.0 (3)	Cl1—C20—H20	109.0
C19—O3—H3A	109.5	Cl3—C20—Cl1	110.5 (3)
C9—N6—C10	101.4 (3)	Cl3—C20—Cl2	109.6 (3)
N4—C9—C8	118.3 (3)	Cl3—C20—H20	109.0
N6—C9—N4	114.1 (3)	Cl2—C20—H20	109.0
N6—C9—C8	127.5 (3)	O1—C17—H17A	109.5
N3—C8—C9	112.1 (3)	O1—C17—H17B	109.5
N3—C8—C7	120.8 (4)	O1—C17—H17C	109.5
C7—C8—C9	127.0 (4)	H17A—C17—H17B	109.5
O1—C14—C13	125.6 (4)	H17A—C17—H17C	109.5
O1—C14—C15	115.7 (3)	H17B—C17—H17C	109.5
C13—C14—C15	118.7 (4)	O3—C19—H19A	109.5
N3—C4—N2	114.2 (3)	O3—C19—H19B	109.5
N3—C4—C5	123.6 (4)	O3—C19—H19C	109.5
C5—C4—N2	122.2 (4)	H19A—C19—H19B	109.5
C11—C16—H16	119.8	H19A—C19—H19C	109.5
C15—C16—H16	119.8	H19B—C19—H19C	109.5
C15—C16—C11	120.5 (4)	O2—C18—H18A	109.5
N2—C3—H3	126.0	O2—C18—H18B	109.5
N2—C3—C2	107.9 (4)	O2—C18—H18C	109.5
C2—C3—H3	126.0	H18A—C18—H18B	109.5
C13—C12—H12	119.3	H18A—C18—H18C	109.5
C13—C12—C11	121.3 (4)	H18B—C18—H18C	109.5
			
Fe1—N3—C8—C9	−0.6 (4)	C9—N6—C10—N5	−1.6 (4)
Fe1—N3—C8—C7	175.9 (3)	C9—N6—C10—C11	177.0 (4)
Fe1—N3—C4—N2	5.5 (5)	C9—C8—C7—C6	175.8 (4)
Fe1—N3—C4—C5	−174.9 (3)	C8—N3—C4—N2	−177.7 (3)
Fe1—N4—N5—C10	−172.7 (3)	C8—N3—C4—C5	1.9 (6)
Fe1—N4—C9—N6	173.7 (3)	C8—C7—C6—C5	0.5 (7)
Fe1—N4—C9—C8	−9.8 (4)	C4—N3—C8—C9	−177.6 (3)
Fe1—N1—C1—C2	175.1 (3)	C4—N3—C8—C7	−1.0 (6)
N3—C8—C7—C6	−0.2 (6)	C4—N2—N1—Fe1	−1.3 (4)
N3—C4—C5—C6	−1.6 (6)	C4—N2—N1—C1	175.1 (3)
O1—C14—C13—C12	−179.6 (4)	C4—N2—C3—C2	−174.5 (4)
O1—C14—C15—O2	−0.2 (5)	C4—C5—C6—C7	0.3 (7)
O1—C14—C15—C16	179.2 (4)	C3—N2—N1—Fe1	−176.9 (3)
N4—N5—C10—N6	1.2 (4)	C3—N2—N1—C1	−0.5 (4)
N4—N5—C10—C11	−177.4 (3)	C3—N2—C4—N3	172.2 (4)
N4—C9—C8—N3	6.9 (5)	C3—N2—C4—C5	−7.4 (7)
N4—C9—C8—C7	−169.4 (4)	C10—N6—C9—N4	1.4 (4)
N2—N1—C1—C2	0.4 (5)	C10—N6—C9—C8	−174.7 (4)
N2—C4—C5—C6	178.0 (4)	C13—C14—C15—O2	−179.3 (4)
N2—C3—C2—C1	−0.2 (5)	C13—C14—C15—C16	0.1 (6)
N5—N4—C9—N6	−0.8 (4)	C13—C12—C11—C16	0.0 (6)
N5—N4—C9—C8	175.8 (3)	C13—C12—C11—C10	−178.2 (4)
N5—C10—C11—C16	17.2 (6)	C11—C16—C15—O2	179.9 (4)
N5—C10—C11—C12	−164.6 (4)	C11—C16—C15—C14	0.5 (6)
N1—N2—C4—N3	−2.5 (5)	C11—C12—C13—C14	0.6 (6)
N1—N2—C4—C5	177.9 (4)	C15—C14—C13—C12	−0.6 (6)
N1—N2—C3—C2	0.4 (5)	C15—C16—C11—C12	−0.6 (6)
N1—C1—C2—C3	−0.2 (5)	C15—C16—C11—C10	177.7 (4)
N6—C9—C8—N3	−177.1 (4)	C17—O1—C14—C13	3.5 (6)
N6—C9—C8—C7	6.6 (7)	C17—O1—C14—C15	−175.5 (4)
N6—C10—C11—C16	−161.3 (4)	C18—O2—C15—C14	179.0 (4)
N6—C10—C11—C12	16.9 (6)	C18—O2—C15—C16	−0.4 (6)
C9—N4—N5—C10	−0.3 (4)		

Symmetry code: (i) −*x*+1, *y*, −*z*+3/2.

### Hydrogen-bond geometry (Å, º)

**Table:**

*D*—H···*A*	*D*—H	H···*A*	*D*···*A*	*D*—H···*A*
C2—H2···C14^ii^	0.95	2.85	3.676 (5)	146
C2—H2···C15^ii^	0.95	2.64	3.585 (5)	171
C7—H7···C1^iii^	0.95	2.81	3.705 (5)	158
C1—H1···N6^iv^	0.95	2.34	3.267 (5)	166
C12—H12···C9^v^	0.95	2.85	3.541 (5)	130
C20—H20···O1	1.00	2.28	3.115 (6)	141
C20—H20···O2	1.00	2.39	3.179 (6)	135
O3—H3*A*···N5	0.84	1.94	2.775 (4)	177
C3—H3···O3^vi^	0.95	2.33	3.238 (5)	161
C5—H5···O3^vi^	0.95	2.47	3.401 (6)	167

Symmetry codes: (ii) −*x*+1, *y*+1, −*z*+3/2; (iii) *x*+1/2, *y*−1/2, −*z*+3/2; (iv) *x*−1/2, *y*+1/2, −*z*+3/2; (v) −*x*+3/2, *y*−1/2, *z*; (vi) *x*+1/2, *y*+1/2, −*z*+3/2.
