# Supplementary material for: Crystal structure of bis­{3-(3,4-di­meth­oxy­phen­yl)-5-[6-(pyrazol-1-yl)pyridin-2-yl]-1,2,4-triazol-3-ato}iron(II)–methanol–chloro­form (1/2/2)
Source: Acta Crystallogr E Crystallogr Commun. 2023 Sep 29;79(Pt 10):962–6. doi: 10.1107/S2056989023008423 (PMC10561207; doi:10.1107/S2056989023008423)
Supplement: Supplementary file 4 [file e-79-00962-sup5.doc]

**Supporting file S1**

**
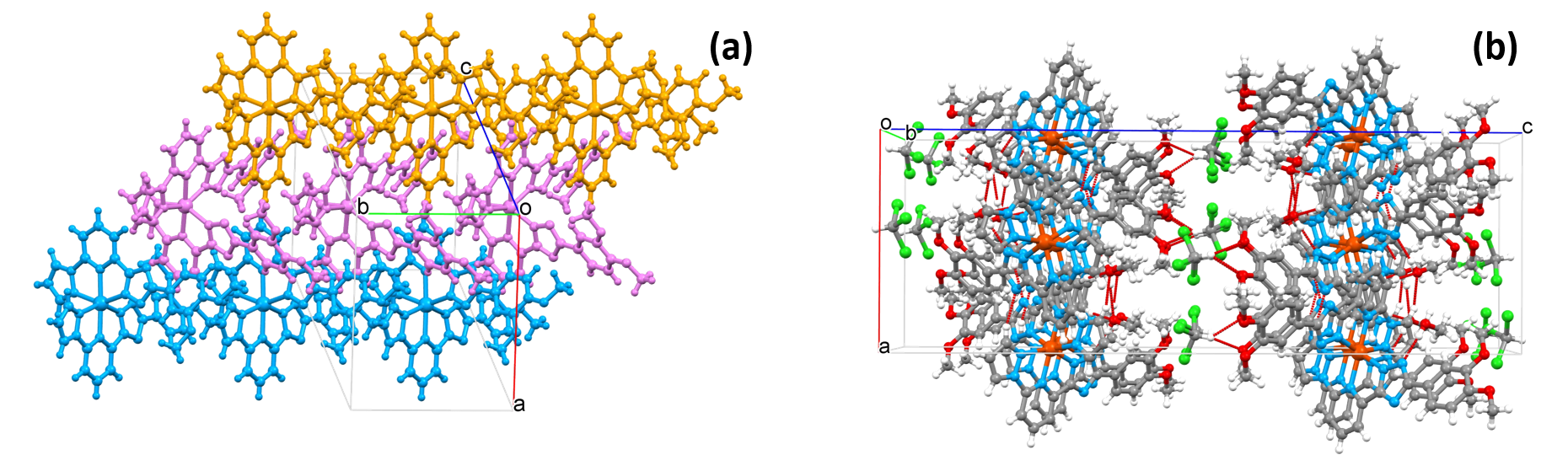
**

**Figure S1.** (a) Two-dimensional layers formed by supramolecular chains. For a better representation, each chain has a different color; (b) Interactions of neighboring layers in a supramolecular network of the title complex. The red dashed lines correspond to the contacts below the sum of van der Waals radii.

**
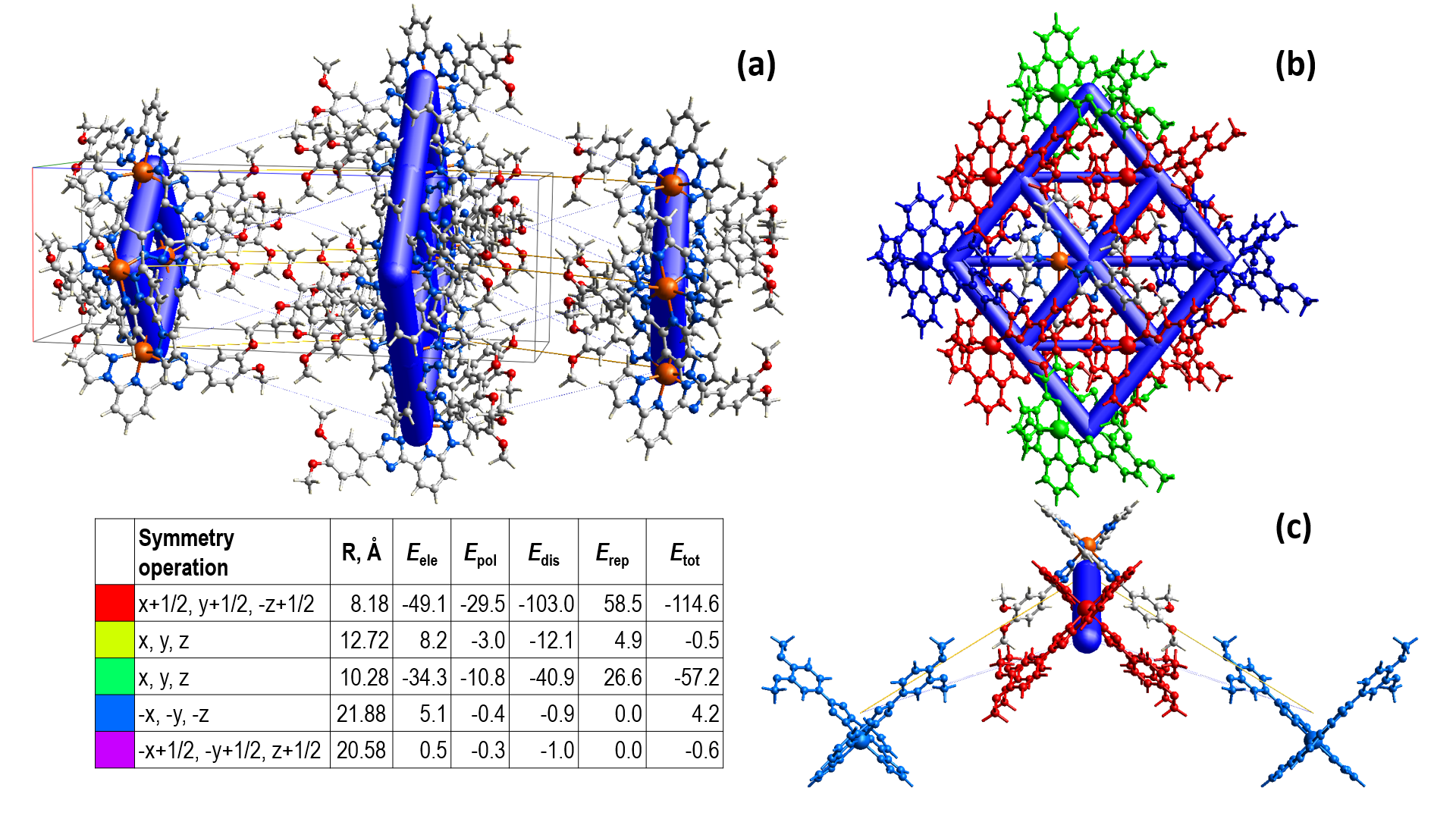
**

**Figure S2.** (a) The calculated energy frameworks, showing the total energy diagrams (*E*_tot_), (b) decomposition of the energy framework into the part corresponding to the interactions within a supramolecular layer and (c) interlayer interactions. In the table the corresponding colour-coded energy values *E*_tot_ are provided, including their E_ele_, *E*_pol_, *E*_dis_, and *E*_rep_ components. Tube size is set at 100 scale, the blue colour corresponds to the attractive interaction, yellow to the repulsive interaction.
